# Chitosan Oligosaccharide Reduces Propofol Requirements and Propofol-Related Side Effects

**DOI:** 10.3390/md14120234

**Published:** 2016-12-21

**Authors:** Zhiwen Li, Xige Yang, Xuesong Song, Haichun Ma, Ping Zhang

**Affiliations:** 1 Department of Anesthesiology, the First Hospital of Jilin University, Changchun 130021, China; li_zhiwen1159@sina.com (Z.L.); yangxige_xg@sina.com (X.Y.); songxs1981@sina.com (X.S.); 2 Department of Hepatobiliary and Pancreatic Surgery, the First Hospital of Jilin University, Changchun 130021, China

**Keywords:** chitosan oligosaccharide, propofol, surgery patients, mouse, dorsal root ganglia, voltage-gated sodium channel gene 1.7

## Abstract

Propofol is one of the main sedatives but its negative side effects limit its clinical application. Chitosan oligosaccharide (COS), a kind of natural product with anti-pain and anti-inflammatory activities, may be a potential adjuvant to propofol use. A total of 94 patients receiving surgeries were evenly and randomly assigned to two groups: 10 mg/kg COS oral administration and/or placebo oral administration before being injected with propofol. The target-controlled infusion of propofol was adjusted to maintain the values of the bispectral index at 50. All patients’ pain was evaluated on a four-point scale and side effects were investigated. To explore the molecular mechanism for the functions of COS in propofol use, a mouse pain model was established. The activities of Nav1.7 were analyzed in dorsal root ganglia (DRG) cells. The results showed that the patients receiving COS pretreatment were likely to require less propofol than the patients pretreated with placebo for maintaining an anesthetic situation (*p* < 0.05). The degrees of injection pain were lower in a COS-pretreated group than in a propofol-pretreated group. The side effects were also more reduced in a COS-treated group than in a placebo-pretreated group. COS reduced the activity of Nav1.7 and its inhibitory function was lost when Nav1.7 was silenced (*p* > 0.05). COS improved propofol performance by affecting Nav1.7 activity. Thus, COS is a potential adjuvant to propofol use in surgical anesthesia.

## 1. Introduction

Propofol (2,6-diisopropylphenol), as a sedative agent, has been used widely in the induction of surgical anesthesia [1]. However, propofol-induced side effects become apparent [2], including hypotension and respiratory depression [3]. Propofol-induced injection pain is a major issue for propofol as an anesthetic in surgery [4,5]. Various alternative and folk remedies have also been used effectively for many years [6,7,8]. Remifentanil preventing propofol-induced injection pain has been proved effective. However, the combination therapy will be affected by the time interval between remifentanil and propofol injection, as well as the dosage of remifentanil [4]. Lidocaine is often used before being injected with propofol. Lidocaine pretreatment or mixed with propofol has also been used successfully for preventing propofol-induced pain [9]. Although the effectiveness is obvious, the side effects of the medicine are also palpable [10,11].

Thus, it is critical to explore a new agent for preventing or treating pain disorders. Chitosan oligosaccharide (COS) is a polysaccharide mainly obtained from crustacean shells and consists of 2-amino-2-deoxy-d-glucan combined with glycoside linkages. COS is made from chitin, which is a homopolymer of 1-4 linked 2-acetamido-2-deoxy-β-d-glucopyranose. COS will be formed when chitin is deacetylated >50%. COS can be applied in many primary industries, including microbial control in agriculture, maintenance of overall fruit and vegetable quality [12] and nutritional dietary additive [13]. Chitosan has many medical and pharmaceutical uses with anti-inflammation and antioxidant activities and fewer side effects [14,15]. The analgesic effect of COS on pain has been proved due to its absorption of proton ions [16]. Thus, COS may be a potential adjuvant to propofol use. To understand the functions of COS, it is necessary to explore the molecular mechanism for the role of COS in propofol therapy.

Voltage-gated sodium channels (Navs) are important indicators of the development of mammalian hyperalgesia [17]. Navs are localized in a mammalian central nervous system [18,19] and DRG (dorsal root ganglia) [20,21]. Navs participate in the pain caused by inflammatory responses [22]. Carrageenan and complete Freund’s adjuvant (CFA) have been used widely to produce mechanical and thermal hyperalgesia in an inflammatory animal model [23,24,25,26]. Thus, these models provide convenient tools in exploring the molecular mechanism of a pain cause. There are many members of Navs with different functions. Three main voltage-gated sodium channels, Nav1.7, Nav1.8, and Nav1.9, are preferentially expressed in dorsal root ganglia (DRG) cells [27]. These channels are involved in different pain. Nav1.9 and Nav1.8 play important roles in the development of cold pain [28]. Previous work showed that little change could be found for inflammation-induced hypersensitivity in the mice lacking Nav1.8 or Nav1.9 [28]. Comparatively, a great reduction in hypersensitivity could be found in Nav1.7 knockout mice [29]. Furthermore, Nav1.7 is essential for burn-induced heat hypersensitivity [30]. An alpha-subunit gene, SCN9A, encodes the Nav1.7 sodium channel [31,32]. An earlier study indicated that SCN9A is essential for human nociception [33]. Sodium channel Nav1.7 is associated with the reduction of neuropathic pain, which is caused by chronic constriction injury of the sciatic nerve in animal models. Behavior tests indicated that the thresholds for thermal and mechanical hyperalgesia were greatly reduced in neuropathic pain models. Meanwhile, the levels of Nav1.7 were significantly increased in DRG cells [34]. In contrast, loss-of-function mutations of Nav1.7 caused congenital insensitivity to pain [35]. Intrathecal injection of Navl.7 shRNA reduced the levels of Nav1.7 and inactivated astrocytes and microglia of DRG. Nav1.7 can improve the pain tolerance in an animal model [36]. Given the key role of Nav1.7 in human pain, the effects of dual therapy on Nav1.7 were investigated.

To uncover the more specific functions of the combined therapy of COS and propofol, the present study was performed to examine the effects of a combined therapy on the level of Nav1.7 in a mouse pain model.

## 2. Results

### 2.1. Chitosan Oligosaccharide (COS) Pretreatment Reduces Propofol Dose during Anesthesia

**Figure 1:**
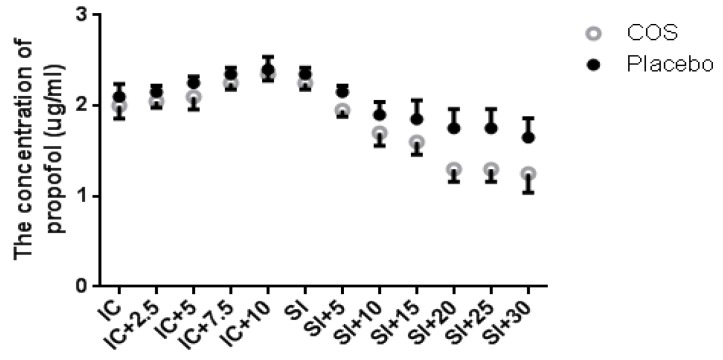
The effects of chitosan oligosaccharide (COS) on propofol requirements. All the selected subjects were evenly assigned to two groups before being injected with propofol: 10 mg/kg COS oral administration and 10 mg/kg placebo oral administration. After five min, propofol was started with step increases of 0.5 μg/mL/2.5 min until the patient lost consciousness. Propofol target-controlled infusion (TCI) was adjusted to maintain the values of bispectral index (BIS) at 50.

According to an earlier study, COS is a kind of hemostatic agent, which can reduce pain by blocking nerve endings [37]. COS pretreatment may prevent propofol-induced injection pain and reduce propofol dose. Thus, the effects of COS on propofol doses were measured. As Figure 1 shows, there was no statistical significance of differences for propofol dose at intubating conditions (*p* > 0.05). In contrast, the effect-site concentration of propofol was greatly lower in CG (COS-pretreated group) than that in PG (placebo-pretreated group) (*p* < 0.05). The results suggest that COS pretreatment reduces propofol dose during anesthesia.

### 2.2. The Incidence of Propofol-Induced Injection Pain in the Subjects Undergoing Surgery

Propofol induces high-incidence pain during intravenous injection. However, few non-pharmacological methods have been applied to control propofol-induced injection pain. COS may be a potential natural product to control the pain. The effects of COS on propofol-induced injection pain were measured. As Table 1 shows, the incidence of propofol-induced pain at a four-point scale in the subjects undergoing surgery was higher in PG than in CG (*p* < 0.05). Furthermore, there was no toxic symptom of COS in all subjects. The results suggest that COS may inhibit the propofol-induced injection pain and can be a potential adjuvant to propofol use.

### 2.3. COS Pretreatment Reduces the Side Effects of Propofol

Besides propofol-induced injection pain, propofol can cause some other side effects. For instance, propofol use induces sedation and may have a significant effect on the pattern of upper airway obstruction [38]. Hypotension has been reported to be a common adverse effect caused by propofol, but there is no reliable method to determine which patients have the risk for propofol-induced hypotension [39]. Therefore, it is necessary to find a new method to control these side effects caused by propofol. Based on this idea, the effects of COS on these side effects were measured. Table 2 shows the most common side effects, which were found in both groups. The patients had lower inadequate ventilation in CG than in PG (*p* < 0.05). Similarly, the patients had a lower incidence of tachycardia and hypotension in CG than in PG (*p* < 0.05). Other side effects showed the similar incidences between two groups. However, there is no statistical significance of differences for bradypnea (*p* > 0.05), and no nausea or vomiting was found in both groups after seven-day surgery, although the symptoms were widely reported in propofol use [40,41].

### 2.4. Analysis of Mechanic Hyperalgesia

**Figure 2:**
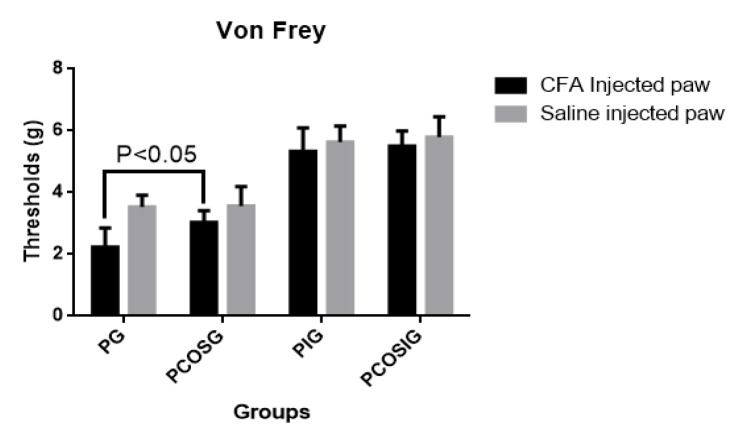
The threshold in an inflammatory pain model. There were 32 mouse pain models evenly assigned into four groups: PG group (received 10 mg/kg propofol treatment), PCOSG group (received both 10 mg/kg COS and propofol treatment), PIG group (Nav1.7-silenced model mouse received 10 mg/kg propofol treatment) and PCOSIG group (Nav1.7-silenced model mouse received both 10 mg/kg COS and propofol treatment). All data were presented as mean ± S.D. and *n* = 8 in each group. There is statistical significance of differences if *p* < 0.05.

Intraplantar injection of 0.9% NaCl solution did not induce mechanical hyperalgesia and is regarded as a control group (Figure 2) Intraplantar injection of CFA increased mechanical hyperalgesia of a mouse model by reducing its thresholds for pain (Figure 2). Propofol and COS treatment decreased CFA-induced hyperalgesia (Figure 2). The combination treatment of COS and propofol attenuated the hyperalgesia more than propofol used alone (*p* < 0.05). However, Nav1.7-silenced groups attenuated hyperalgesia significantly though COS and/or propofol no longer attenuated hyperalgesia (Figure 2). There is no statistical significance of differences among the Nav1.7-silenced groups treated or untreated by COS and/or propofol (*p* > 0.05).

### 2.5. Analysis of Thermal Hyperalgesia

**Figure 3:**
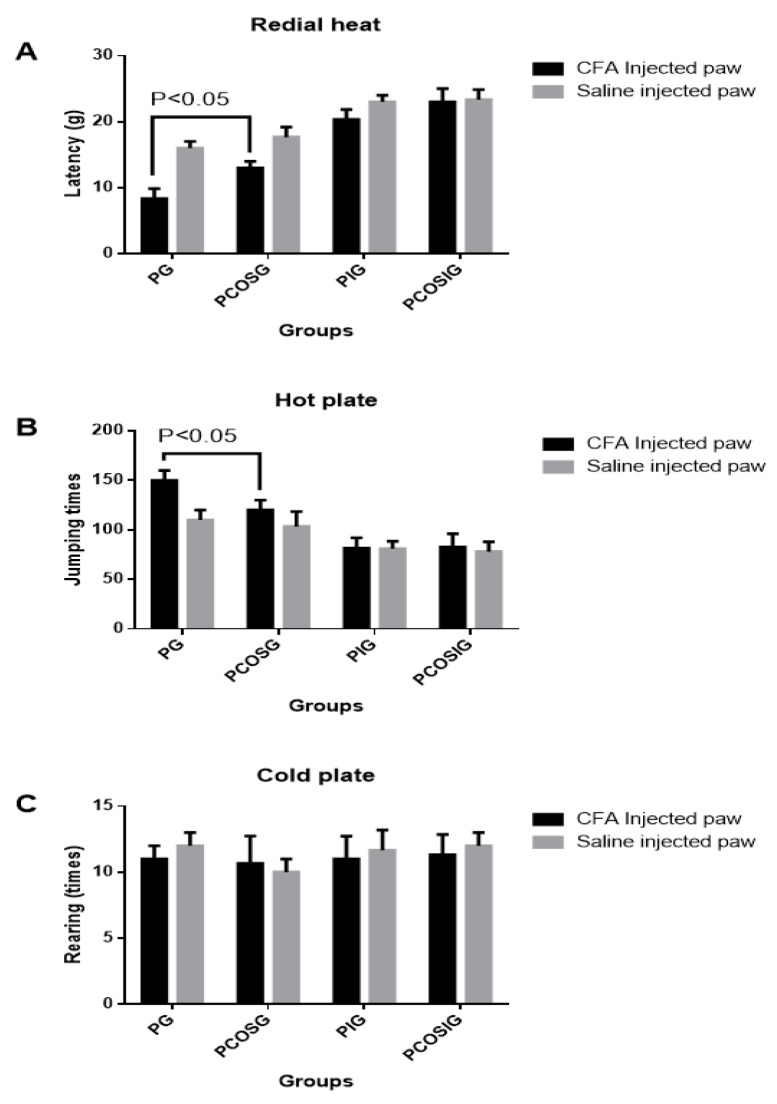
Analysis of hyperalgesia in different groups. (**A**), radial heat analysis of complete Freund’s adjuvant (CFA)-induced thermal hyperalgesia; (**B**), the latency times for jumping responses after exposure to the 50-centigrade plate within 10 min; (**C**), rearing times after exposure to the 50-centigrade plate within 10 min. The mice received 10 mg/kg COS in dietary before 2 h propofol injection. All data were presented as mean ± S.D. and *n* = 8 in each group. There is statistical significance of differences if *p* < 0.05.

Thermal hyperalgesia was found in CFA-induced mouse pain models but not in the mice only treated with 0.9% NaCl solution (Figure 3A). COS reduced thermal hyperalgesia by increasing its latency (Figure 3A). COS attenuated the mechanic hyperalgesia caused by propofol (*p* < 0.05). COS pretreatment resulted in insensitivity to the pain in a mouse model (*p* < 0.05). Comparatively, Nav1.7 silence also attenuated mechanic hyperalgesia significantly but COS no longer attenuated mechanic hyperalgesia (Figure 3A). There is no statistical significance of differences among Nav1.7-silenced groups treated or untreated by COS (*p* > 0.05).

For thermal pain, there is statistical significance of differences for the jumping times between COS-treated and non-treated groups (*p* < 0.05, Figure 3B), suggesting that COS has better effects on thermal hyperalgesia than propofol used alone (*p* < 0.05). Notably, Nav1.7 silence reduced jumping times significantly but propofol and/or COS was not able to reduce jumping times (Figure 3B). There was no statistical significance of differences between the groups treated by propofol and the combination therapy of propofol and COS when Nav1.7 was silenced (*p* > 0.05).

For a cold-plate test, Nav1.7 silence could not reduce the rearing times, and COS and propofol could not maintain reduction of rearing times on the cold plate (Figure 3C). There is no statistical significance of differences among Nav1.7-silenced groups treated or untreated by COS and/or propofol (*p* > 0.05), suggesting that Nav1.7 is also not associated with cold pain.

### 2.6. The Protein Level of Voltage-Gated Sodium Channels (Nav)1.7 in Dorsal Root Ganglia (DRG) Neurons

**Figure 4:**
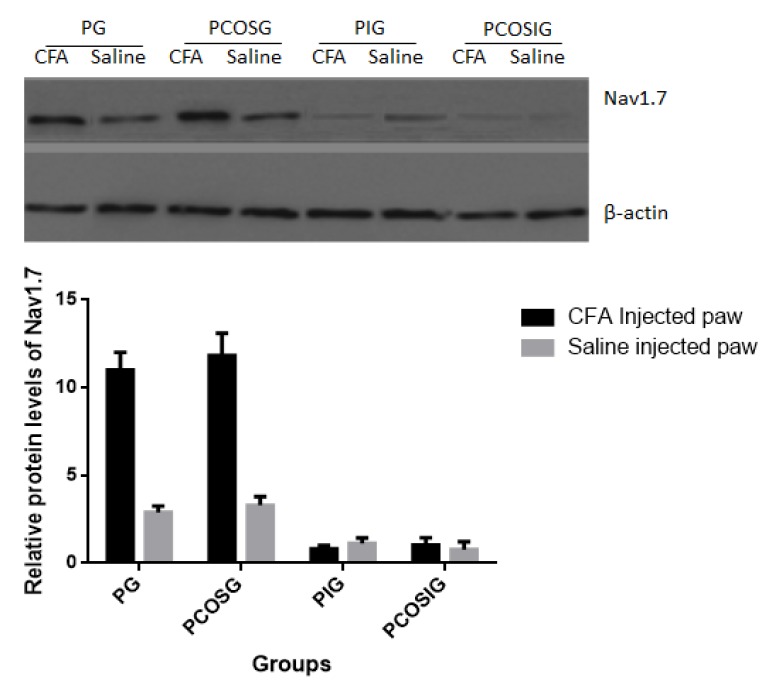
The protein levels of Nav1.7 in the dorsal root ganglia (DRG) neurons of the mice from different groups. All data were presented as mean ± S.D. and *n* = 8 in each group. There is statistical significance of differences if *p* < 0.05.

The protein level of Nav1.7 was analyzed by Western blot. The results showed that Nav1.7 was at a low level when the mice were injected with 0.9% NaCl solution (Figure 4). CFA increased the protein level of Nav1.7 (*p* < 0.01) and there was statistical significance of differences between control and model groups (Figure 4). There was no change in protein level when the mice were treated with propofol and COS (Figure 4) (*p* > 0.05), suggesting that propofol or COS cannot affect the protein level of Nav1.7.

### 2.7. COS Reduces the Activity of Nav1.7

**Figure 5:**
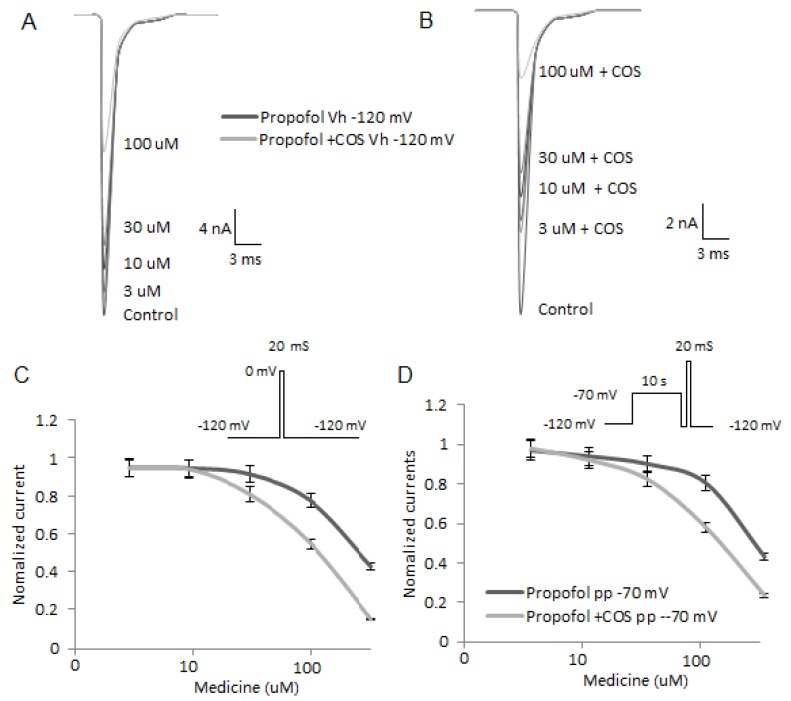
Nav1.7 channels were blocked by propofol and COS. (**A**), Representative traces of Nav1.7 currents in the DRGs treated by different concentrations of propofol. The cells were held at a holding potential of −120 mV and test pulses were stepped to 0 mV and applied at 0.1 Hz; (**B**), Representative traces of Nav1.7 currents in DRGs treated by COS and different concentrations of propofol. The cells were held at a holding potential of −120 mV and test pulses were stepped to 0 mV and applied at 0.1 Hz; (**C**), a tonic block of resting Nav1.7 channels by propofol and/or the combination of COS and propofol. Resting channels were measured at a holding potential of −120 mV; (**D**), a tonic block of inactivated Na+ channels by propofol and/or the combination of COS and propofol. Inactivated channels were induced by a 10 s pre-pulse to −70 mV followed by a 100 ms pulse at −120 mV and a test pulse to 0 mV. Peak amplitudes of Nav1.7 currents were normalized with respect to the peak amplitude in control solution and plotted against the concentration of propofol or a combination of propofol and COS.

To investigate the effects of COS on propofol performance for blocking Nav1.7 activities, the electrophysiological properties of Nav1.7 were compared by using whole-cell patch-clamp recordings. As shown in Figure 5A, propofol blocked Nav1.7 activities in a concentration-dependent manner and COS improved propofol blocking the channels (Figure 5B). Resting channels were measured at a holding potential of −120 mV by test pulses to 0 mV applied at 0.1 Hz. The IC50 values for propofol were 231 ± 12 μM (Hill coefficient 1.8 ± 0.4, *n* = 10) and the values of the combination of COS and propofol were 165 ± 18 μM (Hill coefficient 1.1 ± 0.2, *n* = 10). Figure 5C showed that there was statistical significance of differences for the blocking potencies of resting Na^+^ channels between the propofol and combined groups (*p* = 0.02, unpaired *t*-test). Figure 5D showed that COS enhanced the tonic block of inactivated Na^+^ channels when compared to the group only treated with propofol (propofol, IC50 value 188 ± 10 μM; Hill coefficient 1.6 ± 0.2, *n* = 10; propofol and COS, IC50 value 121 ± 8 μM; Hill coefficient 1.3 ± 0.1, *n* = 10; *p* = 0.02, unpaired *t*-test).

### 2.8. COS Also Promotes Propofol-Produced Stabilization of Fast and Slow Inactivation

**Figure 6:**
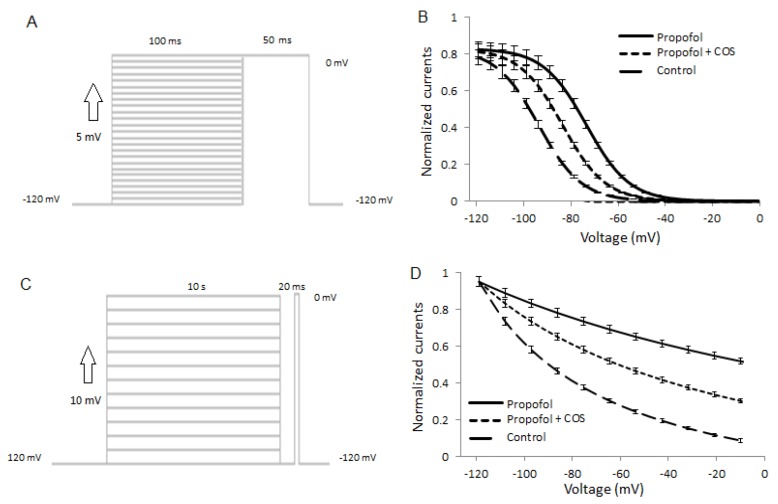
Voltage dependency of fast and slow inactivation of Nav 1.7 in the presence of propofol and COS. (**A**), fast inactivation was caused by 50 ms pre-pulses ranging from −120 to 0 mV in five-mV step, and the remaining fraction of channels was measured with a 20 ms pre-pulse to 0 mV; (**B**), Voltage-dependency of fast inactivation of Nav1.7 in the presence of control solution, or propofol, and/or combination treatment of propofol and COS; (**C**), The voltage protocol of slow inactivation. Slow inactivation was caused by 10 s pre-pulses ranging from −120 to −30 mV in steps of 10 mV followed by a 100-ms interpulse at −120 mV, which allows the recovery from fast inactivation; (**D**), Voltage-dependency of slow inactivation of Nav1.7 in control solution, or propofol, and/or combination treatment of propofol and COS. The lines were fitted by using a Boltzmann equation.

Fast inactivation was caused by 50 ms pre-pulses ranging from −120 to 0 mV in a five-mV step (Figure 6A), and the remaining fraction of channels was measured with a 20 ms pre-pulse to 0 mV. Figure 6B showed that 100 μM propofol caused a ten-mV hyperpolarization shift of steady-state fast inactivation from *V*_1/2_ of −75 ± 2 mV (*n* = 10) in control to *V*_1/2_ of −85 ± 5 mV (*n* = 10) (*p* < 0.01). COS stabilized the fast inactivation and caused a ten-mV hyperpolarization shift of the steady-state fast inactivation of propofol (propofol: *V*_1/2_ of −85 ± 5 mV; propofol and COS: *V*_1/2_ of −95 ± 6 mV; *n* = 10) (*p* < 0.05). There is statistical significance of differences when compared with the combination treatment of COS and propofol (*p* < 0.05). 

Slow inactivation was caused by 10 s pre-pulses ranging from −120 to −10 mV in ten-mV step, followed by a 100 ms pulse at −120 mV, which allows recovery from fast inactivation, and followed by a test pulse to −10 mV. Propofol at 100 μM induced a small shift of the voltage dependency of slow inactivation of Nav1.7 (control: *V*_1/2_ of −20 ± 1 mV; propofol: *V*_1/2_ of −65 ± 2 mV *n* = 10; Figure 6C). In contrast, combination treatment caused the shift of slow inactivation when compared with only propofol used (propofol: *V*_1/2_ of −65 ± 2 mV, combined: *V*_1/2_ of −90 ± 4 mV, *n* = 10; Figure 6C). Neither propofol nor a combination of propofol and COS caused an apparent shift of the voltage-dependency of activation (data not shown).

### 2.9. COS Promotes Propofol Blocking Veratridine-Induced Persistent Sodium Current of Nav1.7

**Figure 7:**
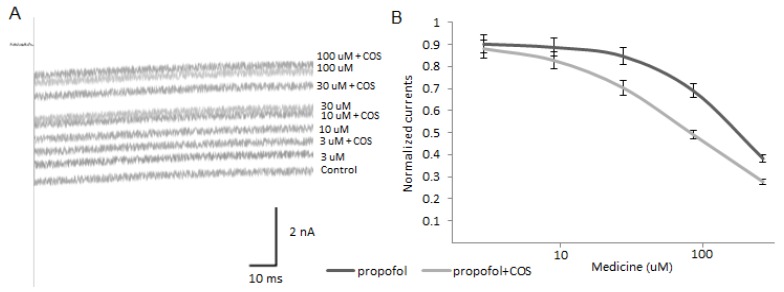
Propofol and COS inhibit persistent Na^+^ currents in DRGs. (**A**), Representative traces of Nav1.7 currents in the presence of 50 μM veratridine. Cells were held at −120 mV and currents were activated at 0.1 Hz; (**B**), the block of the persistent Na^+^ currents in presence of propofol or COS. Peak current amplitudes of the persistent Nav1.7 currents were normalized and fitted with the Hill equation.

To understand the activity of the propofol and the combination of propofol and COS on the persistent Nav1.7 currents, tonic activation was created by adding 50 μM veratridine. Figure 7A shows that veratridine caused a prominent persistent current, which was stimulated by 50 ms pulses in cells at a holding potential of −120 mV. Figure 7B shows that COS promoted propofol blocking the persistent current. The calculated IC50 values of propofol were at 202 ± 27 μM, *n* = 8, and a combination of COS and propofol at 126 ± 47 μM (*p* = 0.03, unpaired *t*-test).

## 3. Discussion

Present findings indicated that COS greatly inhibited the incidence and severity of propofol-induced injection pain if the patients received 10 mg/kg COS via oral administration before being injected with propofol (Table 1). No toxic symptom or fewer side effects were observed in all the patients treated with COS (Table 2). The results suggest that COS may be a potential natural adjuvant to improve propofol performance.

From pain analyses, an animal pain model was successfully established after CFA injection. The mouse model had mechanical and thermal hyperalgesia because of inflammatory pain, which was tested by a von Frey filament assay and hot/cold plate assay. Propofol is one kind of medicine mainly used for decreasing human pain. Present findings indicated that Nav1.7 was increased in CFA-induced hyperalgesia, which suggested that Nav1.7 plays a critical role in inflammatory pain. Subsequent work showed that COS and propofol reduced pain thresholds. 

Injection pain is a normal unwanted adverse effect for propofol use. The side effects can be reduced when combined with COS because they can produce more analgesic efficacy [42]. Another study also used COS as an anesthesia supplement of propofol injection, which was successfully used in topical local anesthesia for surgery on a child [43]. All the results suggest that propofol and COS may have synergistic functions. However, the complementary functions remain unclear. Since many Navs play important roles in pain [44,45] and neural disorders [46,47], we want to explore the effects of combined medicine on the level of Navs. The mutant SCN9A gene-encoding Nav1.7 caused insensitivity to pain in mammals [35]. Furthermore, many pyrrolo-benzo-1,4-diazine derivatives were synthesized to inhibit the activity of Nav1.7, and showed anti-nociceptive oral efficacy in an inflammatory pain model [48]. 

CFA increasing the expression of Nav1.7 was also reported in an earlier study [49]. CFA increased the colocalization of protein kinase B/Akt with Nav1.7 in L4/5 DRG neurons while Akt pathway induced the upregulation of Nav1.7 [50]. Thus, the level of Nav1.7 was higher than in an animal model than in a healthy control. However, no evidence has shown that propofol and COS can reduce the level of Nav1.7 yet (Figure 4). According to a previous report, opioid receptor activation will reduce the level of Nav1.7 [51] while propofol can increase the expression of an opioid receptor [52]. Present work revealed a functional role of COS for controlling pain, which was not associated with the changes of Nav1.7 level (Figure 4). The present findings showed that the combined treatment was better than only one kind of medicine used for decreasing the mechanic and thermal pain (*p* < 0.05)(Figure 2 and Figure 3).

The main aim of our work was to evaluate whether COS and propofol functionally interact with the sodium channel Nav1.7. Our data suggested that COS was a potential adjuvant to improve propofol performance, concentration- and state-dependent inhibitors of Nav1.7. Our results also suggested that propofol and COS interacted and modulated Nav1.7. Therefore, the findings showed that COS reinforced the inhibitory properties of propofol on Nav1.7 activity.

Previous work showed that steady-state plasma concentration of propofol during sedation was in the order of 22–44 μM [53]. It can intensively (97%–98%) bind plasma proteins [54]. In most cases, only the unbound fraction is able to interact with Na^+^ channels. Therefore, a higher concentration was used in pain therapy [55]. Propofol is mainly eliminated by hepatic conjugation to inactive metabolites, which are secreted from the kidney [56]. On the other hand, the persons have a reduced clearance for propofol and may have increased levels of plasma propofol [57]. Additionally, the terminal half-life of propofol ranges from one to three days [58].

**Figure 8:**
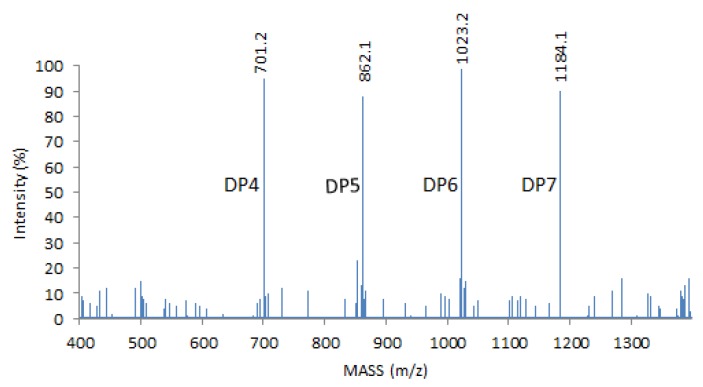
MALDI–TOF MS analysis of COS. The main products for the degree of polymerization (DP) were DP4, 5, 6 and 7 when potassium adducts ions were calculated in MALDI-TOF.

COS showed as a preventive agent by improving propofol performance in a pain model. COS improves propofol performance by suppressing pain symptoms and inhibiting Nav1.7 activity (Figure 6, Figure 7 and Figure 8). Furthermore, COS caused an obvious hyperpolarization shift of the steady-state fast inactivation of Nav1.7 (Figure 6). There is statistical significance of differences when compared to the combination of COS and propofol (*p* < 0.05, unpaired *t*-test). COS has no systemic adverse effects on the mouse model. Clinically relevant plasma levels of propofol will cause related effects on Nav1.7. Therapeutic levels of COS are low in the present experiment (10 mg/Kg).

One important thing should be mentioned here: −120 mV hyperpolarized potentials were artificial and did not present the membrane properties of DRGs in vivo. With a physiological resting membrane potential around −50 mV, and with an ongoing DRG activity, the data from inactivated channels can be used to evaluate the function of Na^+^ channel blockers. A tonic block of Nav1.7 channels by propofol and COS may be a better means for pain therapy. Present findings showed that COS were potential adjuvants to induce a higher tonic block as compared to use of only propofol. 

There are some limitations for the present study: (1) Most studies, if not all, examined the effect of COS in addition to propofol, and the possible effects of COS alone have not been studied. This seems to make the mechanisms of COS effects vague and mysterious. Propofol has been proved to be an important sedative. However, we are not sure whether only COS can be a kind of sedative although it has been reported to have anti-pain functions. To avoid unknown risks, the test was not performed in the patients receiving surgeries. We are influenced by the design for human experiment and the test was not performed in the animal models with only COS treatment; (2) Low-molecular-weight COS cannot be injected in most cases although it has been used widely as healthy products in China; (3) Detail molecular mechanism for the inhibitory function of COS and propofol for Nav1.7 remains unknown; (4) Nav1.7 is only one critical effector for evaluating the functions of COS, and many other Nav members should be analyzed in the future.

## 4. Materials and Methods

### 4.1. COS Preparation and MALDI–TOF (Matrix-Assisted Laser-Desorption Ionization–Time-of-Flight) MS Analysis

Low-molecular-weight, water-soluble COS was purchased from GlycoBio Company (Dalian, China). The COS was marine natural products and prepared from marine resources according to a previous report [59]. A 1 μL sample solution was mixed with 2 μL 2,5-dihydroxybenzoic acid (15 mg/mL) in 30% ethanol. Mass spectra were made on an Agilent 6530 Accurate-Mass (Santa Clara, CA, USA) in a positive ion mode. In the measurement, a nitrogen laser (Spectra-Physics, Mountain View, CA, USA) (at 337 nm, 3 ns pulse width, 3 Hz) was performed. All spectra were examined in a reflector mode by using external calibration. MALDI–TOF MS analysis of COS showed that the degree of polymerization (DP) of the main products were DP4, 5, 6 and 7 when potassium adduct ions were summed together in MALDI-TOF (Figure 8).

### 4.2. Participants

Before the present study, all protocols were approved by the Ethical Committee of the First Hospital of Jilin University (Changchun, China). The subjects with the physical status of American Association of Anesthesiology (ASA) I or II received surgery at our hospital from 3 May to 12 October. Including criteria was used according to previously reported [60]. Excluding criteria includes following items: (1) the patients could not express themselves clearly; (2) they took other anti-pain medicine within one day of surgery; (3) the patients refused to sign an informed consent for present experiments. Finally, a total of 188 patients were selected.

### 4.3. Patient Grouping

All the selected subjects were evenly assigned to two groups before being injected with propofol: 10 mg/kg COS (CG) treatment and 10 mg/kg placebo (PG) treatment daily. COS and placebo were administered orally. To avoid the intervention of baseline characters for final results, demographic data were investigated including age, gender, BMI (body mass index), lifestyle and ASA. After 2 h pretreatment, the patients received 2 mg/kg/h saline treatment. After five min, propofol TCI was started with step increases of 0.5 μg/mL/2.5 min until the patient lost consciousness. *Cis*-atracurium was injected at 0.2 mg/kg to promote tracheal intubation. Meanwhile, propofol TCI was adjusted to maintain BIS values at 50. The pain was evaluated by clinical experts according to a four-point scale (no pain, mild pain, moderate pain and severe pain) from propofol injection to the time when the patients lost consciousness. Side effects were recorded from day 4 to 7 after the surgery. Table 3 showed that the baseline characters were similar between CG and PG groups, including age, gender, BMI, lifestyle and ASA (*p* < 0.05). The results suggest that the baseline clinical characters will not affect the final results of COS and propofol treatment.

### 4.4. Animals

To explore the molecular mechanism, an animal pain model was established. All the protocols were established according to the guidance for the use of laboratory animals (National Academy Press) and approved by the Ethical Committee of the First Hospital of Jilin University (Changchun, China). Four-week-old C57BL/6 male mice were purchased from Shanghai SLAC Laboratory Animal Co., Ltd. (Shanghai, China). A total of 32 mice (20–25 g) were anesthetized with 2% isoflurane (Cat. No. CDS019936, Sigma, St. Louis, MO, USA) and injected with complete Freund’s adjuvant (CFA, Cat. No. F5881, 10 μL 0.5 mg/mL heat-killed *M. tuberculosis*) (Sigma, St. Louis, MO, USA) in the plantar of one hind paw to cause inflammatory pain symptoms. Meanwhile, another hind paw was injected with 10 μL 0.9% NaCl as a control. Animal behaviors were observed after one-day pain induction.

### 4.5. Nav1.7 Gene Silencing

pTZU6+1 vector was from Chongqing Medical University (Chongqing, China). shRNA for Nav1.7 gene silencing was constructed by using the primers: sense, 5′-ACCTCGACCTCAGAGCTTCGTTCACTTTGGAGTGAACGAAGCTCTGAGGTCTT-3’; antisense, 5’-CAAAAAGACCTCAGAGCTTCGTTCACTCCAAAGTGAACGAAGCTCTGAGGTCG-3′. Restriction sites, *Sal*I and *Xba*I, were added on either end of the oligos and linked with pTZU6+1, and pTZU6+1-Nav1.7 were reconstructed. The reconstructed plasmids were injected into mice via tail veins. Eight hours after injection, propofol injection was performed and animal behaviors of mechanical and thermal hyperalgesia were analyzed.

### 4.6. Animal Grouping

The mice received 10 mg/kg COS treatment before 2 h propofol injection and the dosage was used according to a previous report [61]. There were 32 pain-model mice evenly assigned into four groups: PG group (received 10 mg/kg propofol treatment), PCOSG group (received both 10 mg/kg COS and propofol treatment), PIG group (Nav1.7-silenced model mouse received 10 mg/kg propofol treatment) and PCOSIG group (Nav1.7-silenced model mouse received both 10 mg/kg COS and propofol treatment).

### 4.7. Animal Behavior of Mechanical and Thermal Hyperalgesia

Mechanic pain sensitivity was measured immediately by testing the responding forces to the stimulation by Electronic von Frey monofilaments (Nanjing Jisheng Medical Technology Company, Nanjing, China) after propofol injections. The thermal pain was examined by an algesiometer (Shanghai AoBopharmtech, Shanghai, China). Hot- and cold-induced pains were tested by a Hot/Cold Plate Analgesia Meter (YLS-6B, Huaibei Zhenghua Biologic Apparatus Facilities Ltd. Co., Huaibei, China).

### 4.8. Western Blot

According to a previous report, CFA infection increases the expression of Nav1.7 [49]. Nav1.7 can be upregulated in L4/5 DRG neurons in a certain evoking situation [50]. Therefore, L4-5 DRG samples from different groups were obtained. Protein was isolated using a plasma membrane protein isolation kit (Cat. No. ab65400, Abcam Trading (Shanghai) Company Ltd., Shanghai, China). Rabbit anti-mouse monoclonal Nav1.7 antibody (Cat. No. 62758, dilution 1:5000, Abcam Trading (Shanghai) Company Ltd., Shanghai, China) was used as the first antibody. Polyclonal Goat Anti-Rabbit IgG H&L (Cat. No. ab6721, dilution 1:3000, Abcam Trading (Shanghai) Company Ltd., Shanghai, China) was used as a secondary antibody. A rabbit anti-mouse β-actin polyclonal antibody (1:2000 dilution; Cat. No. 4967, Cell Signaling Technology, Danvers, MA, USA) was used as a loading control. All protein bands were visualized by using an enhanced chemiluminescence substrate (Sangon Biotech, Co., Ltd., Shanghai, China). The image intensity of protein bands was quantified by using NIH ImageJ software (Bethesda, MD, USA).

### 4.9. Electrophysiology Analysis of Nav1.7

Primary DRG cells were cultured in DMEM media and treated with different concentrations of propofol and/or 10 μg/mL COS for 24 h. To investigate the activities of Nav1.7, the electrophysiological properties of Nav1.7 were compared in primary DRG cells by using whole-cell patch-clamp recordings. The following test solution was prepared (mM): 100 NaCl, 50 choline chloride, 5 KCl, 1 MgCl_2_, 1 CaCl_2_, 10 HEPES, and 15 glucose. The pH value was adjusted to 7.0 with tetraethylammonium hydroxide (Sigma, St. Louis, MO, USA). The pipette solution consists of the following components (mM): 140 CsF, 10 NaCl, 1 ethyleneglycol-bis (2-aminoethylether)-*N*,*N*,*N*′,*N*′-tetraacetic acid, 10 HEPES and pH value was adjusted to 7.0 with CsOH.

The membrane currents were recorded by using a patch clamp and an EPC10 amplifier (HEKA Instruments Inc., Bellmore, NY, USA). Data were obtained and stored with Patchmaster v20 × 60 software (HEKA Instruments Inc., Bellmore, NY, USA). Patch pipettes were pulled from glass capillaries (Science Products, Hofheim, Germany) by using a DMZ-Universal Puller (Zeitz, Germany) and then heat polished to give a resistance of 2.0 to 2.5 MΩ when it was filled with pipette solution. Currents were filtered at 5 kHz. The series resistance was compensated by 60%–80% to minimize voltage errors, and the capacitance artifacts were canceled using the amplifier circuitry. Linear leak subtraction based on resistance estimates from hyperpolarized pulses was applied before the pulse test.

### 4.10. Statistical Analysis

M Data were represented as mean ± S.D. Chi-square test was used for the comparison between two groups. The comparisons of independent groups of data were performed with the ANOVA test by using IBM SPSS Statistics 20.0 (Brea, CA, USA). Data analysis, curve fitting, and statistical analyses were also performed using the same software. IC50 values were calculated by normalizing peak current amplitudes at different concentrations to the value obtained in control solution. Data were fitted with Hill equation *y* = *y*_max_ × (IV50*n*/IC50*n* × C*n*), where *y*_max_ is the maximal amplitude, IC50 is the concentration at which *y*/*y*_max_ = 0.5, and *n* is the Hill coefficient. To obtain inactivation curves, peak currents evoked by a test pulse were measured, normalized, and plotted against the conditioning repulse potential. Data were fitted by the Boltzmann equation [62], *y* = 1/(1 + exp(Epp − h0.5)/kh), where Epp is the membrane potential of test pulse, h0.5 is the voltage at which *y* equals 0.5, and kh is a slope factor.

## 5. Conclusions

Taken together, propofol and chitosan oligosaccharide (COS) can synergistically reduce inflammation pain symptoms. While propofol causes some adverse effects, COS improves the propofol performance with fewer side effects by reducing inflammation and inhibiting the activity of voltage-gated sodium channel (Nav)1.7. Our data demonstrate that both substances block the Na^+^ channel Nav1.7 and potentially contribute to pain relief. Thus, this study identified a potential adjuvant for the pain therapy with low-dose propofol.

## Figures and Tables

**Table 1:** Intravenous COS pretreatment reduces propofol-induced pain.

Grade, *n* (%)	CG (*n* = 47)	PG (*n* = 47)	Chi-Square Statistic	*p* Values
No pain	36 (76.6)	6 (12.77)	39.25	0.000
Mild pain	4 (8.51)	12 (25.53)		
Moderate pain	5 (10.64)	16 (34.04)		
Severe pain	2 (4.26)	13 (27.66)		
Total pain	11 (23.4)	41 (87.23)	38.736	0.000

Note: Chi-square test was performed. 4 × 4 contingency test was used for the comparison of four-grade pain and 2 × 2 contingency test was used for the comparison of total pain. BMI, body mass index. There is statistical significance of differences if *p* < 0.05.

**Table 2:** The effects of COS on the side effects caused by propofol.

Side Effects	CG (*n* = 47)	PG (*n* = 47)	Chi-Square Statistic	*p* Values
Apnea	2 (4.26)	8 (17.02)	2.798	0.094
Bradypnea (breaths < 6/min)	1 (2.13)	6 (12.77)	2.470	0.116
Obstructive respiration	0 (0)	5 (10.64)	4.451	0.035
Tachycardia (HR > 30% above BL)	1 (2.13)	8 (17.02)	4.424	0.035
Hypertension(MAP > 30%above BL)	0 (0)	6 (12.77)	2.470	0.116
Bradycardia (HR > 30% under BL)	0 (2.13)	5 (10.64)	4.451	0.035
Hypotension (MAP > 30% under BL)	0 (0)	7 (14.89)	5.557	0.018
Burning and stinging	2 (4.26)	10 (21.28)	6.114	0.013

Note: Chi-square test was performed. HR: heart rate, MAP: mean arterial pressure, BL = baseline (measurement before induction). There is statistical significance of differences if *p* < 0.05.

**Table 3:** Baseline characters of patients receiving surgery between CG and PG groups.

Baseline Characters	CG (*n* = 47)	PG (*n* = 47)	T Value/Chi-Square Statistic	*p* Values
Age	38.9 ± 15.6	40.1 ± 16.8	0.141	0.235
Gender, male (%)	32 (68.09)	34 (72.34)	0.203	0.652
BMI	22.7 ± 4.8	23.9 ± 6.5	0.037	0.326
Smoking, *n* (%)	28 (59.57)	26 (55.32)	0.174	0.677
Drinking, *n* (%)	29 (61.7)	25 (53.19)	0.696	0.404
Spouse, *n* (%)	44 (93.62)	46 (97.87)	0.261	0.409
ASA				
I	35 (74.47)	37 (78.72)	0.237	0.626
II	12 (25.53)	10 (21.28)		

Note: *t*-test and Chi-squared test were performed. CG, the patients received COS oral administration before being injected with propofol. PG, the patients received placebo oral administration before being injected with propofol. ASA, American Society of Anesthesiologists. There is statistical significance of differences if *p* < 0.05.

## References

[1] Sapate M., Andurkar U., Markandeya M., Gore R., Thatte W. (2015). To study the effect of injection dexmedetomidine for prevention of pain due to propofol injection and to compare it with injection lignocaine. Braz. J. Anesthesiol..

[2] Marik P.E. (2004). Propofol: Therapeutic indications and side-effects. Curr. Pharm. Des..

[3] Mays N. (2011). Reducing unwarranted variations in healthcare in the English NHS. BMJ.

[4] Lee S.H., Lee S.E., Chung S., Lee H.J., Jeong S. (2016). Impact of time interval between remifentanil and propofol on propofol injection pain. J. Clin. Anesth..

[5] Madan H.K., Singh R., Sodhi G.S. (2016). Comparsion of Intravenous Lignocaine, Tramadol and Keterolac for Attenuation of Propofol Injection Pain. J. Clin. Diagn. Res..

[6] Berberian P., Obimba C., Glickman-Simon R., Sethi T. (2015). Herbs for Low-Back Pain, Acupuncture for Psychological Distress, Osteopathic Manipulative Therapy for Chronic Migraine, Honey Dressings for Burns, Vegetarian Diet and Risk of Colorectal Cancer. Explore.

[7] Schroder S., Beckmann K., Franconi G., Meyer-Hamme G., Friedemann T., Greten H.J., Rostock M., Efferth T. (2013). Can medical herbs stimulate regeneration or neuroprotection and treat neuropathic pain in chemotherapy-induced peripheral neuropathy?. Evid. Based Complement. Altern. Med..

[8] Tatsumi S., Mabuchi T., Abe T., Xu L., Minami T., Ito S. (2004). Analgesic effect of extracts of Chinese medicinal herbs Moutan cortex and Coicis semen on neuropathic pain in mice. Neurosci. Lett..

[9] Euasobhon P., Dej-Arkom S., Siriussawakul A., Muangman S., Sriraj W., Pattanittum P., Lumbiganon P. (2016). Lidocaine for reducing propofol-induced pain on induction of anaesthesia in adults. Cochrane Database Syst. Rev..

[10] Joo J.D., In J.H., Kim D.W., Jung H.S., Kang J.H., Yeom J.H., Choi J.W. (2012). The comparison of sedation quality, side effect and recovery profiles on different dosage of remifentanil patient-controlled sedation during breast biopsy surgery. Korean J. Anesthesiol..

[11] McCleskey P.E., Patel S.M., Mansalis K.A., Elam A.L., Kinsley T.R. (2013). Serum lidocaine levels and cutaneous side effects after application of 23% lidocaine 7% tetracaine ointment to the face. Dermatol. Surg..

[12] Romanazzi G., Feliziani E., Banos S.B., Sivakumar D. (2017). Shelf life extension of fresh fruit and vegetables by chitosan treatment. Crit. Rev. Food Sci. Nutr..

[13] Swiatkiewicz S., Swiatkiewicz M., Arczewska-Wlosek A., Jozefiak D. (2015). Chitosan and its oligosaccharide derivatives (chito-oligosaccharides) as feed supplements in poultry and swine nutrition. J. Anim. Physiol. Anim. Nutr. (Berl.).

[14] Chung M.J., Park J.K., Park Y.I. (2012). Anti-inflammatory effects of low-molecular weight chitosan oligosaccharides in IgE–antigen complex-stimulated RBL-2H3 cells and asthma model mice. Int. Immunopharmacol..

[15] Guo M., Ma Y., Wang C., Liu H., Li Q., Fei M. (2015). Synthesis, anti-oxidant activity, and biodegradability of a novel recombinant polysaccharide derived from chitosan and lactose. Carbohydr. Polym..

[16] Aranaz I., Mengíbar M., Harris R., Paños I., Miralles B., Acosta N., Galed G., Heras Á. (2009). Functional characterization of chitin and chitosan. Curr. Chem. Biol..

[17] Lai J., Porreca F., Hunter J.C., Gold M.S. (2004). Voltage-gated sodium channels and hyperalgesia. Annu. Rev. Pharmacol. Toxicol..

[18] Shah B.S., Stevens E.B., Pinnock R.D., Dixon A.K., Lee K. (2001). Developmental expression of the novel voltage-gated sodium channel auxiliary subunit β3, in rat CNS. J. Physiol..

[19] Whitaker W., Faull R., Waldvogel H., Plumpton C., Burbidge S., Emson P., Clare J. (1999). Localization of the type VI voltage-gated sodium channel protein in human CNS. Neuroreport.

[20] Yin R., Liu D., Chhoa M., Li C.M., Luo Y., Zhang M., Lehto S.G., Immke D.C., Moyer B.D. (2015). Voltage-gated sodium channel function and expression in injured and uninjured rat dorsal root ganglia neurons. Int. J. Neurosci..

[21] Rabert D.K., Koch B.D., Ilnicka M., Obernolte R.A., Naylor S.L., Herman R.C., Eglen R.M., Hunter J.C., Sangameswaran L. (1998). A tetrodotoxin-resistant voltage-gated sodium channel from human dorsal root ganglia, hPN3/SCN10A. Pain.

[22] Cohen C.J. (2011). Targeting voltage-gated sodium channels for treating neuropathic and inflammatory pain. Curr. Pharm. Biotechnol..

[23] Suh H.R., Chung H.J., Park E.H., Moon S.W., Park S.J., Park C.W., Kim Y.I., Han H.C. (2015). The effects of Chamaecyparis obtusa essential oil on pain-related behavior and expression of pro-inflammatory cytokines in carrageenan-induced arthritis in rats. Biosci. Biotechnol. Biochem..

[24] Yang Y., Li Y.X., Wang H.L., Jin S.J., Zhou R., Qiao H.Q., Du J., Wu J., Zhao C.J., Niu Y. (2015). Oxysophocarpine Ameliorates Carrageenan-induced Inflammatory Pain via Inhibiting Expressions of Prostaglandin E2 and Cytokines in Mice. Planta Med..

[25] Jiang Y.L., He X.F., Shen Y.F., Yin X.H., Du J.Y., Liang Y.I., Fang J.Q. (2015). Analgesic roles of peripheral intrinsic met-enkephalin and dynorphin A in long-lasting inflammatory pain induced by complete Freund’s adjuvant in rats. Exp. Ther. Med..

[26] Qian B., Li F., Zhao L.X., Dong Y.L., Gao Y.J., Zhang Z.J. (2015). Ligustilide Ameliorates Inflammatory Pain and Inhibits TLR4 Upregulation in Spinal Astrocytes Following Complete Freund’s Adjuvant Peripheral Injection. Cell. Mol. Neurobiol..

[27] Strickland I.T., Martindale J.C., Woodhams P.L., Reeve A.J., Chessell I.P., McQueen D.S. (2008). Changes in the expression of NaV1.7, NaV1.8 and NaV1.9 in a distinct population of dorsal root ganglia innervating the rat knee joint in a model of chronic inflammatory joint pain. Eur. J. Pain.

[28] Leo S., D’Hooge R., Meert T. (2010). Exploring the role of nociceptor-specific sodium channels in pain transmission using Nav1.8 and Nav1.9 knockout mice. Behav. Brain Res..

[29] Nassar M.A., Stirling L.C., Forlani G., Baker M.D., Matthews E.A., Dickenson A.H., Wood J.N. (2004). Nociceptor-specific gene deletion reveals a major role for Nav1.7 (PN1) in acute and inflammatory pain. Proc. Natl. Acad. Sci. USA.

[30] Shields S.D., Cheng X., Üçeyler N., Sommer C., Dib-Hajj S.D., Waxman S.G. (2012). Sodium channel Nav1.7 is essential for lowering heat pain threshold after burn injury. J. Neurosci..

[31] De Rooij A.M., Gosso M.F., Alsina-Sanchis E., Marinus J., Hilten J.J.V., Maagdenberg A.M.V.D. (2010). No mutations in the voltage-gated NaV1.7 sodium channel alpha1 subunit gene SCN9A in familial complex regional pain syndrome. Eur. J. Neurol..

[32] Diss J.K., Calissano M., Gascoyne D., Djamgoz M.B., Latchman D.S. (2008). Identification and characterization of the promoter region of the Nav1.7 voltage-gated sodium channel gene (SCN9A). Mol. Cell. Neurosci..

[33] Cox J.J., Reimann F., Nicholas A.K., Thornton G., Roberts E., Springell K., Karbani G., Jafri H., Mannan J., Raashid Y. (2006). An SCN9A channelopathy causes congenital inability to experience pain. Nature.

[34] Liu C., Cao J., Ren X., Zang W. (2012). Nav1.7 protein and mRNA expression in the dorsal root ganglia of rats with chronic neuropathic pain. Neural Regen. Res..

[35] Minett M.S., Pereira V., Sikandar S., Matsuyama A., Lolignier S., Kanellopoulos A.H., Mancini F., Iannetti G.D., Bogdanov Y.D., Santana-Varela S. (2015). Endogenous opioids contribute to insensitivity to pain in humans and mice lacking sodium channel Nav1.7. Nat. Commun..

[36] Gandini R., Merolla S., Chegai F., Del Giudice C., Stefanini M., Pampana E. (2015). Foot Embolization During Limb Salvage Procedures in Critical Limb Ischemia Patients Successfully Managed With Mechanical Thromboaspiration: A Technical Note. J. Endovasc. Ther..

[37] SudheesháKumar P. (2014). Flexible, micro-porous chitosan–gelatin hydrogel/nanofibrin composite bandages for treating burn wounds. RSC Adv..

[38] Capasso R., Rosa T., Tsou D.Y., Nekhendzy V., Drover D., Collins J., Zaghi S., Camacho M. (2016). Variable Findings for Drug-Induced Sleep Endoscopy in Obstructive Sleep Apnea with Propofol versus Dexmedetomidine. Otolaryngol. Head Neck Surg..

[39] Au A.K., Steinberg D., Thom C., Shirazi M., Papanagnou D., Ku B.S., Fields J.M. (2016). Ultrasound measurement of inferior vena cava collapse predicts propofol-induced hypotension. Am. J. Emerg. Med..

[40] Bang Y.S., Kim Y.U., Oh D., Shin E.Y., Park S.K. (2016). A randomized, double-blind trial evaluating the efficacy of palonosetron with total intravenous anesthesia using propofol and remifentanil for the prevention of postoperative nausea and vomiting after gynecologic surgery. J. Anesth..

[41] Bataille A., Letourneulx J.F., Charmeau A., Lemedioni P., Leger P., Chazot T., Guen M.L., Diemunsch P., Fischler M., Liu N. (2016). Impact of prophylactic combination of dexamethasone-ondansetron on postoperative nausea and vomiting in obese adult patients undergoing laparoscopic sleeve gastrectomy during closed-loop propofol-remifentanil anaesthesia: A randomised double-blind placebo study. Eur. J. Anaesthesiol..

[42] Cho S.Y., Jeong C.W., Jeong C.Y., Lee H.G. (2010). Efficacy of the combination of cold propofol and pretreatment with remifentail on propofol injection pain. Korean J. Anesthesiol..

[43] Zhang J., Wang Y., Li B., Zhang W. (2010). Remifentail infusion for paediatric bronchoscopic foreign body removal: Comparison of sevoflurane with propofol for anaesthesia supplementation for bronchoscope insertion. Anaesth. Intensive Care.

[44] Rivara M., Zuliani V. (2015). Novel sodium channel antagonists in the treatment of neuropathic pain. Expert Opin. Investig. Drugs.

[45] Hockley J.R., Winchester W.J., Bulmer D.C. (2016). The voltage-gated sodium channel Na 1.9 in visceral pain. Neurogastroenterol. Motil..

[46] Mackenzie F.E., Parker A., Parkinson N.J., Oliver P.L., Brooker D., Underhill P., Lukashkina V.A., Lukashkin A.N., Holmes C., Brown S.D. (2009). Analysis of the mouse mutant Cloth-ears shows a role for the voltage-gated sodium channel Scn8a in peripheral neural hearing loss. Genes Brain Behav..

[47] Teixeira C.E., Baracat J.S., Arantes E.C., de Nucci G., Antunes E. (2005). Effects of β-adrenoceptor antagonists in the neural nitric oxide release induced by electrical field stimulation and sodium channel activators in the rabbit corpus cavernosum. Eur. J. Pharmacol..

[48] Yang S.W., Ho G.D., Tulshian D., Bercovici A., Tan Z., Hanisak J., Brumfield S., Matasi J., Sun X., Sakwa S.A. (2014). Bioavailable pyrrolo-benzo-1,4-diazines as Nav 1.7 sodium channel blockers for the treatment of pain. Bioorg. Med. Chem. Lett..

[49] Huang C.-P., Chen H.-N., Su H.-L., Hsieh C.-L., Chen W.-H., Lai Z.-R., Lin Y.-W. (2013). Electroacupuncture reduces carrageenan-and CFA-induced inflammatory pain accompanied by changing the expression of Nav1.7 and Nav1.8, rather than Nav1.9, in mice dorsal root ganglia. Evid. Based Complement. Altern. Med..

[50] Liang L., Fan L., Tao B., Yaster M., Tao Y.-X. (2013). Protein kinase B/Akt is required for complete Freund’s adjuvant-induced upregulation of Nav1.7 and Nav1.8 in primary sensory neurons. J. Pain.

[51] Chattopadhyay M., Mata M., Fink D.J. (2008). Continuous delta-opioid receptor activation reduces neuronal voltage-gated sodium channel (NaV1.7) levels through activation of protein kinase C in painful diabetic neuropathy. J. Neurosci..

[52] Li Z., Pei Q., Cao L., Xu L., Zhang B., Liu S. (2012). Propofol increases micro-opioid receptor expression in SH-SY5Y human neuroblastoma cells. Mol. Med. Rep..

[53] Richards M.J., Skues M.A., Jarvis A.P., Prys-Roberts C. (1990). Total i.v. anaesthesia with propofol and alfentanil: Dose requirements for propofol and the effect of premedication with clonidine. Br. J. Anaesth..

[54] Altmayer P., Buch U., Buch H.P. (1995). Propofol binding to human blood proteins. Arzneimittelforschung.

[55] Ludbrook G.L., Upton R.N., Grant C., Gray E.C. (1996). Brain and blood concentrations of propofol after rapid intravenous injection in sheep, and their relationships to cerebral effects. Anaesth. Intensive Care.

[56] Raoof A.A., Obbergh L.J.V., de Goyet J.V., Verbeeck R.K. (1996). Extrahepatic glucuronidation of propofol in man: Possible contribution of gut wall and kidney. Eur. J. Clin. Pharmacol..

[57] Shafer A., Doze V.A., Shafer S.L., White P.F. (1988). Pharmacokinetics and pharmacodynamics of propofol infusions during general anesthesia. Anesthesiology.

[58] Kotani Y., Shimazawa M., Yoshimura S., Iwama T., Hara H. (2008). The experimental and clinical pharmacology of propofol, an anesthetic agent with neuroprotective properties. CNS Neurosci. Ther..

[59] Younes I., Rinaudo M. (2015). Chitin and chitosan preparation from marine sources. Structure, properties and applications. Mar. Drugs.

[60] Becher R.D., Peitzman A.B., Sperry J.L., Gallaher J.R., Neff L.P., Sun Y., Miller P.R., Chang M.C. (2016). Damage control operations in non-trauma patients: Defining criteria for the staged rapid source control laparotomy in emergency general surgery. World J. Emerg. Surg..

[61] Yousef M., Pichyangkura R., Soodvilai S., Chatsudthipong V., Muanprasat C. (2012). Chitosan oligosaccharide as potential therapy of inflammatory bowel disease: Therapeutic efficacy and possible mechanisms of action. Pharmacol. Res..

[62] Wang S.Y., Wang G.K. (1997). A mutation in segment I-S6 alters slow inactivation of sodium channels. Biophys. J..

